# A collagen membrane influences bone turnover marker in vivo after bone augmentation with xenogenic bone

**DOI:** 10.1186/s13005-020-00249-9

**Published:** 2020-12-07

**Authors:** Henning Staedt, Michael Dau, Eik Schiegnitz, Daniel G. E. Thiem, Olga Tagadiuc, Victor Palarie, Peter Ottl, Bilal Al-Nawas, Peer W. Kämmerer

**Affiliations:** 1 Private Practice, Esslingen am Neckr, Germany; 2 Department of Prosthodontics and Materials Science, University Medical Center Rostock, Strempelstraße 13, 18057 Rostock, Germany; 3 Department of Oral, Maxillofacial Plastic Surgery, University Medical Center Rostock, Schillingallee 35, 18057 Rostock, Germany; 4 Department of Oral, Maxillofacial Plastic Surgery, University Medical Center Mainz, Augustusplatz 2, 55131 Mainz, Germany; 5 Laboratory of Biochemistry, State University of Medicine and Pharmacy “Nicolae Testemitanu”, Stefan cel Mare si Sfant Boulevard 165, 2004 Chisinau, Moldova; 6 Laboratory of Tissue Engineering and Cell Cultures, State University of Medicine and Pharmacy “Nicolae Testemitanu”, Stefan cel Mare si Sfant Boulevard 165, 2004 Chisinau, Moldova

**Keywords:** Bone regeneration, Membrane, Collagen, Bone remodeling, Serological bone turnover markers, Animal study

## Abstract

**Background:**

The aim was to compare early biochemical and histological osseous healing of chronic mandibular defects regenerated with bovine bone substitute with and without collagen membrane in vivo.

**Methods:**

Eight weeks after formation of a lateral full-thickness perforating bone defect in the mandible of 40 rabbits, bovine bone substitute with (“+”;*n* = 20) and without (“-”;*n* = 20) collagen membrane was applied. Blood and bone was collected 24, 72 h, 7, 14 and 21 days after surgery. Total acid phosphatase, bone acid phosphatase, total alkaline phosphatase and bone alkaline phosphatase activities were compared between groups. Formation of new bone was quantified histologically for all time points.

**Results:**

Twenty-four hours after surgery, bone alkaline phosphatase was significantly elevated in “+” group when compared to “-” (p=0.012). After 72 hours, all bone turnover markers except for total acid phosphatase (p=0.078) where significantly elevated in “+” (all *p* < 0.05). Fourteen days after surgery, the significant highest values for all bone turnover markers were detected in “-” (all *p* < 0.05). A significant difference in favor of group “-” could also be detected after 3 weeks in terms of both acid phosphatases (*p* < 0.05). In histology, no significant differences could be detected.

**Conclusion:**

Bone regeneration with bovine bone substitute material and collagen membrane shows a significantly earlier bone remodeling activity but does not seem to influence formation of new bone in histological samples.

## Background

Guided bone regeneration (GBR) is an established treatment method for regeneration of osseous defects of the jaws. GBR is based on a bone graft material and a barrier membrane to cover and stabilize the augmented bone defect [1–3]. Membranes for GBR can be divided into non-resorbable and resorbable. Non-resorbable systems include titanium meshes and polytetrafluoroethylene membranes. Those materials have to be removed in a second procedure after completion of the regenerative bone healing [4], whereas absorbable membranes are dissolved by catalytic processes [2, 5]. These membranes mostly consist of porcine or bovine collagen and are characterized by a good barrier function [3, 6]. As a biomaterial, collagen has a number of properties including low immunogenicity, fast vascularization, promotion of wound healing and even bone regeneration [7–11]. It was shown that the material allows sufficient diffusion of nutrients for cellular proliferation and differentiation [12]. Deproteinized bovine bone is a well-documented natural hydroxyapatite that promotes bone healing and implant osseointegration during osseous regeneration procedures in the jaws [3, 13–17]. Besides, it has shown to be resistant to resorption [18]. The combination of resorbable collagen membranes with underlying bovine bone substitute material successfully led to osseous regeneration in animal models as well as in human jaws [3, 19–21].

Radiological examination is the standard method for estimating the osseous healing and consolidation process after augmentation. However, correlations between healing status and callus resistance are assumed to be very low [22]. Bone turnover markers are products of bone cell activity, have a prognostic value for early detection of osseous healing [22, 23] and might be an interesting alternative for evaluation of graft remodeling. Bone turnover markers can be divided into bone resorption and formation markers. Tartrate-resistant acid phosphatase is mainly present in osteoclasts and - to a lesser extent - in osteoblasts and osteocytes [24–27]. Alkaline phosphatase is attached to the outer surface of cells and matrix vesicles and has an important role in development and mineralization of bone. In brief, acid phosphatase may facilitate bone mineralization in the osteocyte lacunae and alkalic phosphatase positively influences osteoblast-derived mineralization. Both bone remodeling enzymes exhibit significant activity versus the mineralization inhibitor inorganic pyrophosphate and regulate osteopontin, that could inhibit de novo bone formation [24, 28]. However, there are no studies examining activity of bone turnover markers after bone augmentation procedures in the jaws so far.

Therefore, this experimental project was designed to evaluate differences in the sequential osseous healing events that occur during early stages of bone regeneration in mandibular chronic lateral ridge defects in rabbits using bovine bone substitute material covered or non-covered with a collagen membrane. The outcomes of bone regeneration were measured by the activities of acid phosphatase and alkaline phosphatase in peripheral blood and within bone as well as by histological measurement of new formed bone within the augmented defect.

## Methods

### Study materials

Deproteinized bovine bone substitute material (BBSM; Bio-Oss; Geistlich Pharma AG, Wolhusen, Switzerland; granularity 1–2 mm), as well as resorbable, non-cross-linked collagen membranes (Bio-Gide; Geistlich Pharma AG, Wolhusen, Switzerland) were used. BBSM is deproteinized bovine cancellous bone with a structure similar to human bone and osteoconductive characteristics. In brief, it consists of a natural, non-antigenic, porous bone mineral matrix and it is produced by removal of all organic components from bovine bone [3, 29]. The porcine-derived type I and III collagen membrane has a bilayer structure, consisting of a compact outer layer and a porous inner layer of collagen fiber bundles [10]. Both materials, alone and in combination by means of GBR-procedures, are frequently used in preclinical as well as clinical regenerative maxillofacial surgery [3, 18, 30–32].

### Experimental animal model

**Fig. 1:**
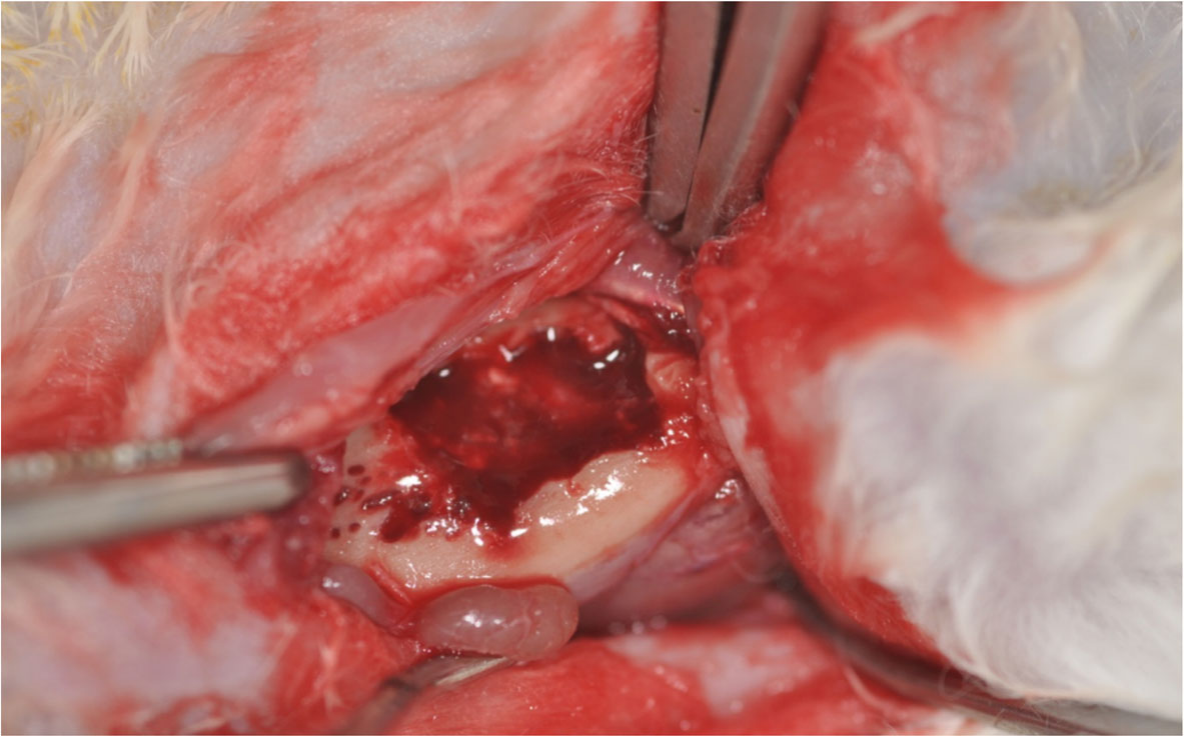
Bone defect in the rabbit mandible before bone grafting procedure

The study was planned prospectively in accordance to the ARRIVE guidelines [33] and the EU Directive 2010/63/EU for animal experiments. The animal experiments were approved by the Research Ethics Committee for Laboratory Animals at the University of Medicine and Pharmacy “Nicolae Testemitanu”, Chisinau, Moldova. Fourty, 9 months old, 4–5 kg, female New Zealand white rabbits were used for the in vivo experiments. All animals were treated in accordance with both policies and principles of laboratory animal care and with the European Union guidelines. The rabbits were housed in individual cages in an animal room maintained at 22 °C and 55% relative humidity with ventilation 18–20 times/h and a 12-h light–dark cycle. They were allowed free access to diet and water. The treatment consisted of two surgical approaches under general anesthesia (intramuscular injections of a combination of a dose of 35 mg/kg body weight ketamine and a dose of 5 mg/kg body weight xylazine) each. Prior to any surgical intervention, local anesthetic was applied (4% articaine with 1:200.000 epinephrine (Ultracaine DS, Sanofi, Frankfurt am Main, Germany)) followed by disinfection using chlorhexidine (Chlorhexamed FORTE 0.2%, GlaxoSmithKline Consumer Healthcare, Bühl, Germany). At the first surgical step, a full thickness critical size perforating bone defect removing both cortical plates and the trabecular bone [34] (1 × 1 cm) was created at the right side of the mandible in all animals after incision of the skin and elevation of the periosteum. The wounds were closed with absorbable sutures (Vicryl 4–0 (Ethicon GmbH, Norderstedt, Germany)). Eight weeks after formation of the defect, the regeneration procedure was carried out. In brief, using the same surgical approach as in the first step, the bone was carefully skimmed with a straight fissure carbide bur under copious irrigation with sterile 0.9% physiological saline to remove remaining soft tissue and to lay open fresh bone tissue (Fig. 1). In no defect, osseos healing was seen and each defect was augmented using BBSM. In a randomized approach using a computerized list, the animals either received a collagen membrane to cover the BBSM-containing defect (group +; *n* = 20) or none such membrane (group -; *n* = 20). The membranes were put under the periosteum and no further fixation was conducted. The mucoperiosteal flaps, muscles, subcutaneous tissue and skin were advanced, repositioned anatomically and fixed via interrupted and mattress sutures with Vicryl 4–0. Postoperatively, Ibuprofen (2–10 mg/kg body weight orally) was used for analgesia.

### Measurements

#### Bone turnover marker

**Fig. 2:**
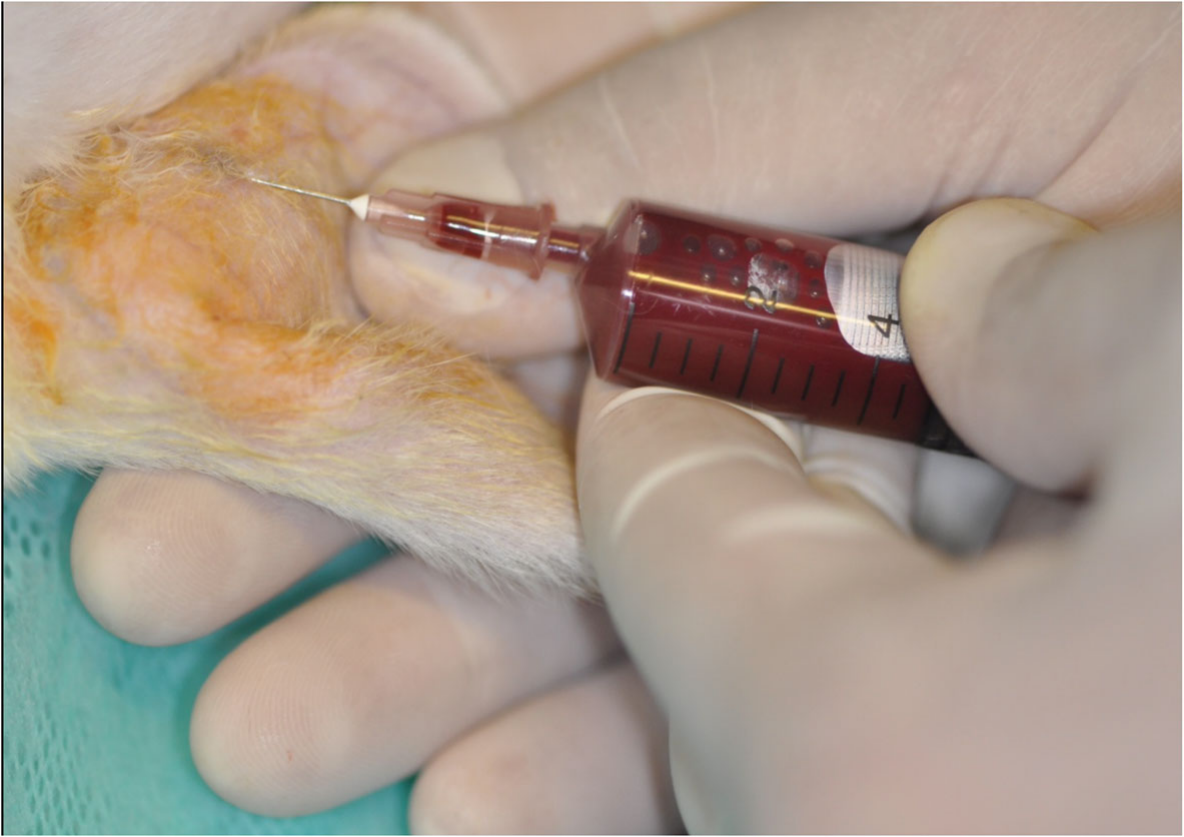
Collection of peripheral blood from the ear vein of a rabbit

The outcomes of bone regeneration were measured by activities of total acid phosphatase out of the peripheral blood (TAcPh; Fig. 2), bone acid phosphatase out of the ground bone from the augmented site (BAcPh), total alkaline phosphatase (TAlPh) as well as bone alkaline phosphatase (BAlPh). The peripheral blood (TAcPh; (TAlPh) as well as the bone (BAcPh; BAlPh) samples were collected immediately after 24 and 72 h, 7 days, 2 weeks and 3 weeks after surgery with *n* = 4 samples per group. Blood samples were obtained from anesthetized animals before sacrifice. Animals were sacrificed with an intravenous overdose of pentobarbital (100 mg/kg body weight). Half of the augmented sites were removed *en bloc*, ground into particles and processed for enzyme analysis, using standard kits and following the manufacturer’s protocol (Acid Phosphatase Assay Kit CS0740, Sigma-Aldrich, Taufkirchen, Germany; Alkaline Phosphatase Detection Kit AFP, Sigma-Aldrich, Taufkirchen, Germany). The serum was separated by centrifugation at 3000 g for 10 min at 37 °C. Aliquots were stored at 80 °C in appropriate cuvettes.

#### Histology

Using the other half of the samples, histological analysis on formation of new bone was carried out. For this purpose, following fixation in 4% buffered formaldehyde and dehydration, the specimens were embedded in a 1:1 combination of glycol-methacrylate and ultraviolet light-activated polymethyl-methacrylate (Technovit 7200® VLC; Heraeus Kulzer, Hanau, Germany) for 5 days. After penetration of the whole specimen by Technovit 7200® VLC, the slides were carefully photopolymerized and processed applying the sawing and grinding technique [35] by using the microgrinding system EMS (Exact, Norderstedt, Germany) to a thickness of 10–20 μm and stained with toluidine blue. Finally, five representative cut and ground sections from the core for each defect were digitized using a color scanner with a resolution of 2400 dpi. Additionally, an empty histology slide with millimeter scale was scanned for calibration. The pictures were digitally edited with imaging software (Adobe Photoshop CS, Adobe Systems Software Ireland Ltd., Dublin, Ireland) in order to amplify the contrast between remaining bone graft material and soft tissue by color-coding. The slides were evaluated histomorphometrically using the computer software Analysis® (Soft-Imaging-Systems, Münster, Germany). For each slide, the amount of new-formed bone within the augmented matrix (%) was analyzed as described before [16]. For each defect, mean values were created out of 3 slides and used for further calculations. All measurements were therefore performed in triplicates. Examiners were blinded to the kind of augmentation.

#### Statistics

The study was carried out as a pilot study as there was no prior analysis on enzymatic activities after guided bone regeneration procedures in the literature. The case number of *n* = 4 per time point and group is comparable to other animal studies reporting GBR-procedures [3, 36, 37]. Comparisons were conducted between the membrane groups and between the different time periods for each group. A non-parametric Kruskal–Wallis test was used to identify statistical differences between the experimental groups or the time points. Whenever a statistical difference was found, the Mann–Whitney test was applied. Analyses were made using SPSS Version 24 software (SPSS, Inc., Chicago, USA) and the significance level was set at *p* < 0.05. The data are presented as the mean ± standard deviation. *P*-values < 0.05 were described as “statistically significant”, although no adjustment for multiple tests has been applied and the *p*-values are reported descriptively only.

## Results

The post-operative healing was generally uneventful. All animals completed the study and could be included in the descriptive statistical analysis. No complications such as fractures, allergic reactions, swellings, abscesses, or infections were noticed throughout the entire study period.

### Bone turnover marker

**Fig. 3:**
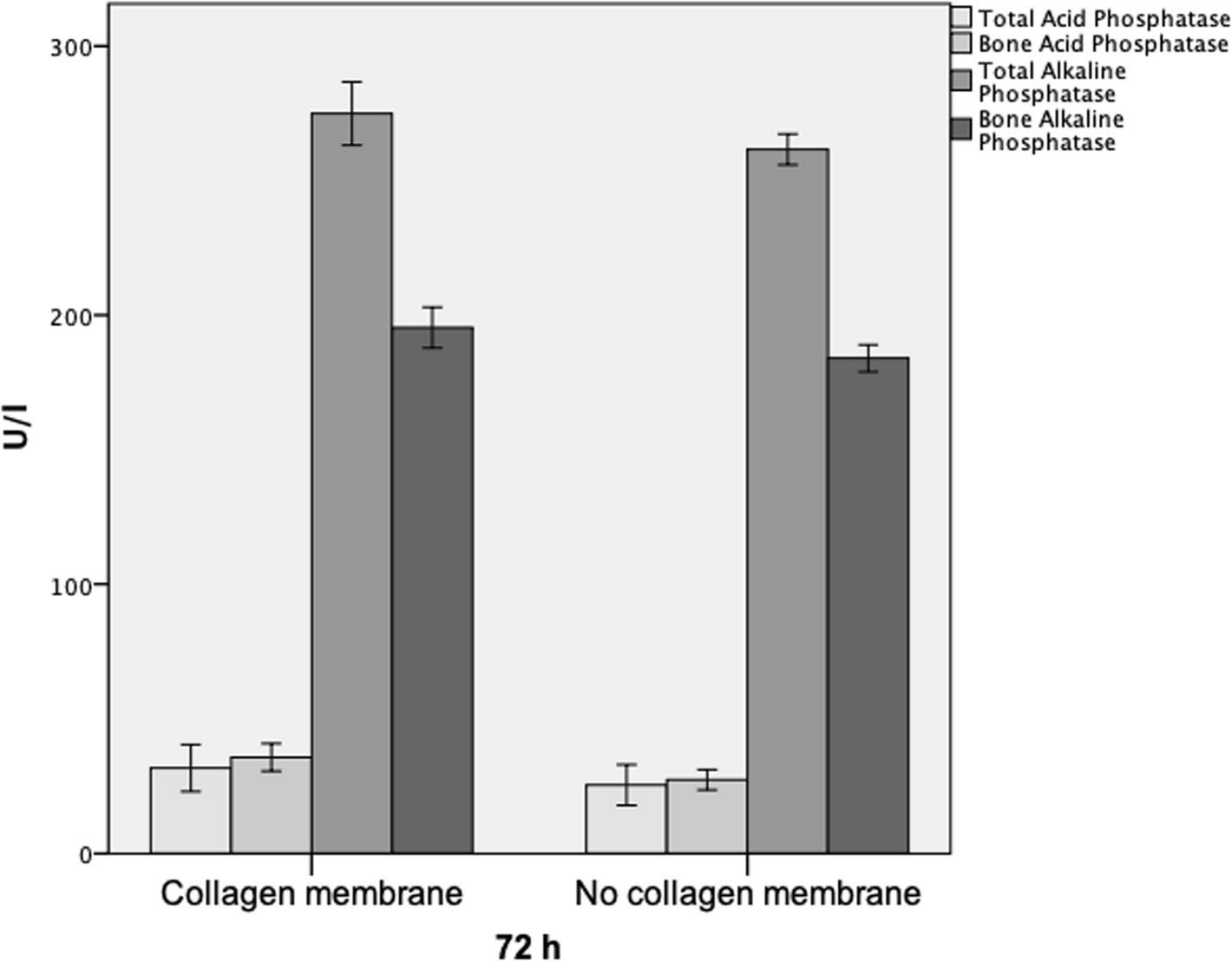
Bar charts showing expression of all four bone remodeling markers after 72 h. Significant differences in favor of the group with collagen membrane were detected in case of bone acid phosphatase, as well as total and bone alkalic phosphatase (all *p* < 0.05)

**Fig. 4:**
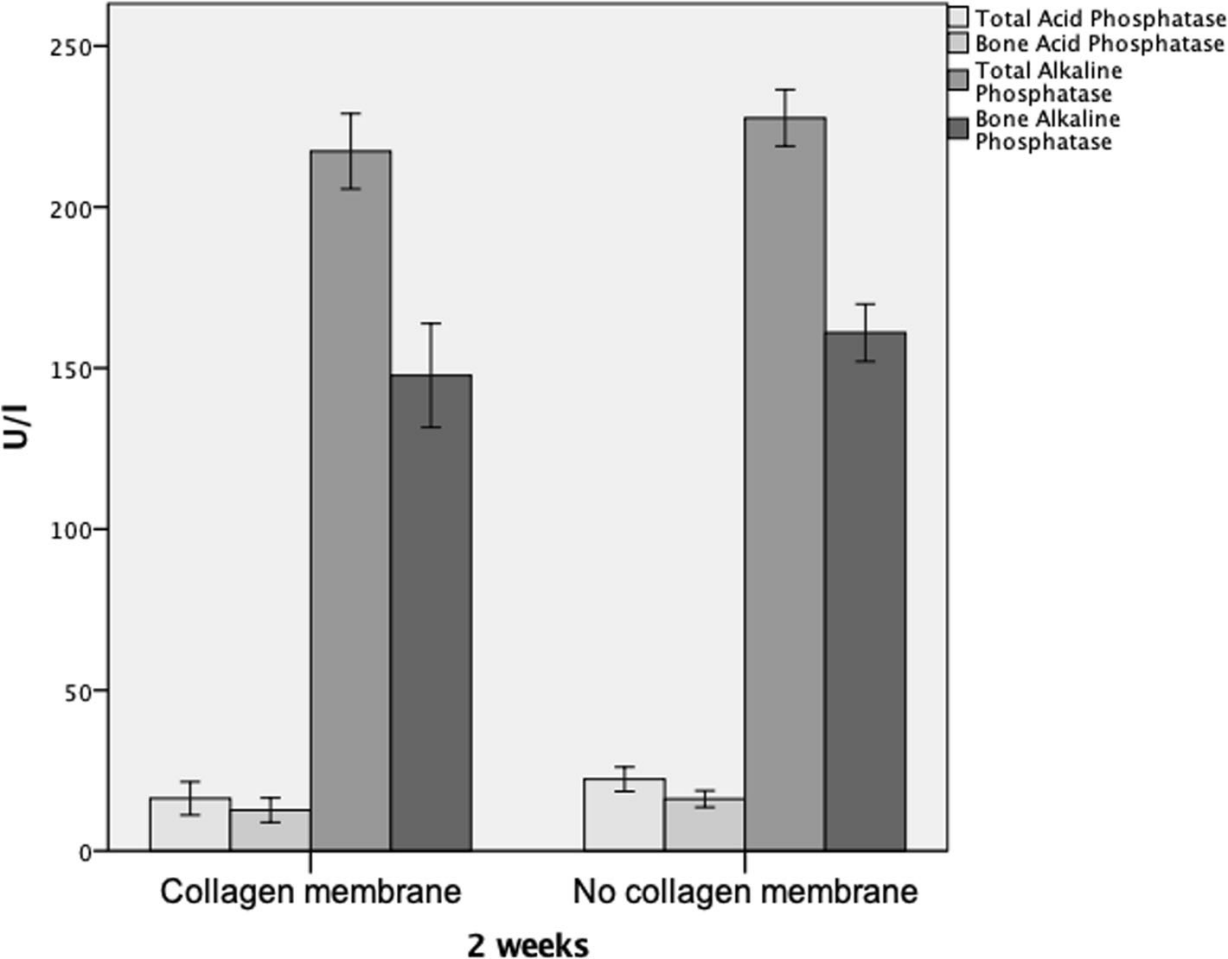
Bar charts showing expression of all four bone remodeling markers after 2 weeks. Significant differences in favor of the non-collagen group were detected in case of all four bone remodeling markers (all *p* < 0.05)

Twenty-four hours after surgery, bone alkaline phosphatase (BAlPh) was significantly elevated in the collagen (+) group when compared to the non-collagen samples (*p* = 0.012; Table 1). After 72 h, all bone turnover markers except for TAcPh (*p* = 0.078) where significantly elevated in the group treated with collagen membranes (BAcPh: *p* = 0.005, TAlPh: *p* = 0.013, BAlPh: *p* = 0.06; Table 1, Fig. 3). Seven days after surgery, none such differences were seen anymore (Table 2). Fourteen days after surgery, the significant highest values for all bone turnover markers were detected in the group without collagen membrane (TAcPh: *p* = 0.016, BAcPh: *p* = 0.031, TAlPh: *p* = 0.038, BAlPh: *p* = 0.036; Table 2, Fig. 4). A significant difference in favor of group “-” could also be detected after 3 weeks in terms of acid phosphatase (TAcPh: *p* = 0.01, BAcPH: *p* = 0.026; Table 2).

**Table 1:**
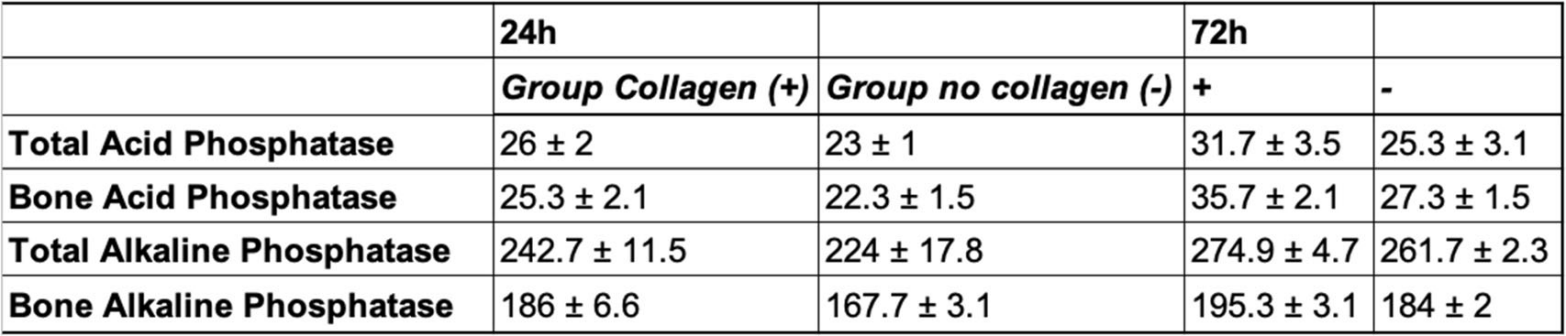
Mean values as well as standard deviations for all bone turnover markers at24 h and 72 h after augmentation of the defect.

**Table 2:**
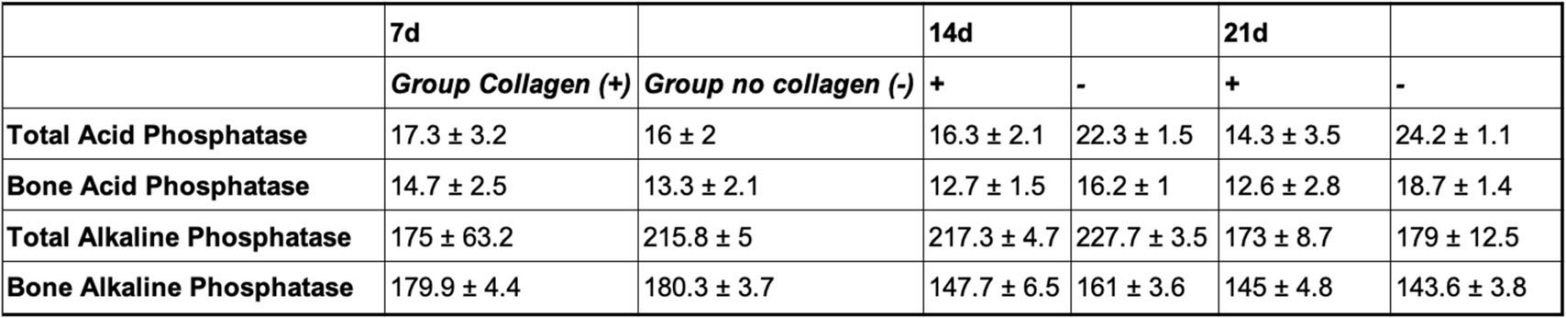
Mean values as well as standard deviations for all bone turnover markers 7 d, 14 d and 21 d after augmentation of the defect.

### Histology

**Fig. 5:**
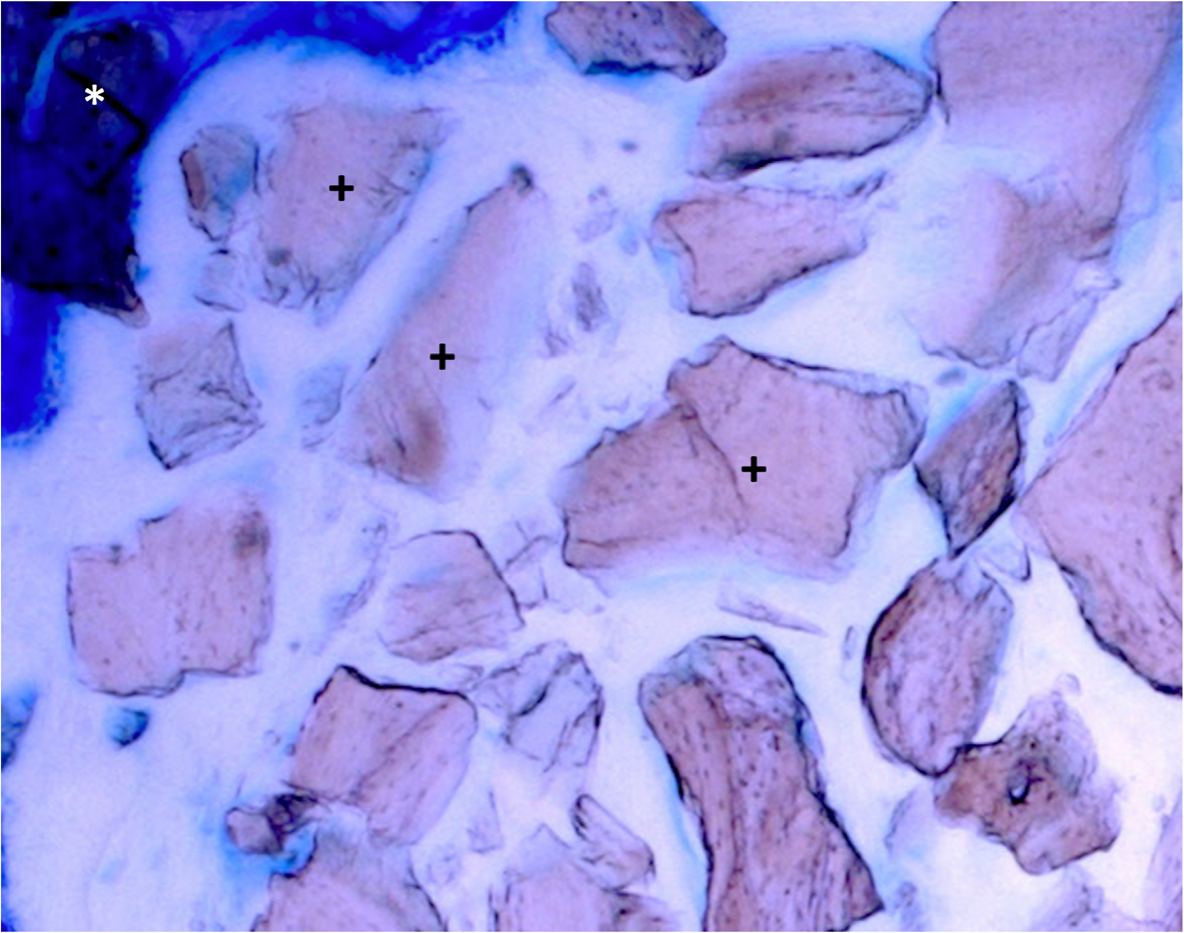
Histology (toluidine blue, original magnification × 20) of a specimen (group with collagen membrane) after 7 days. No new bone formation can be seen. * = residual bone at the edge of the defect; + = bovine bone substitute particles

**Fig. 6:**
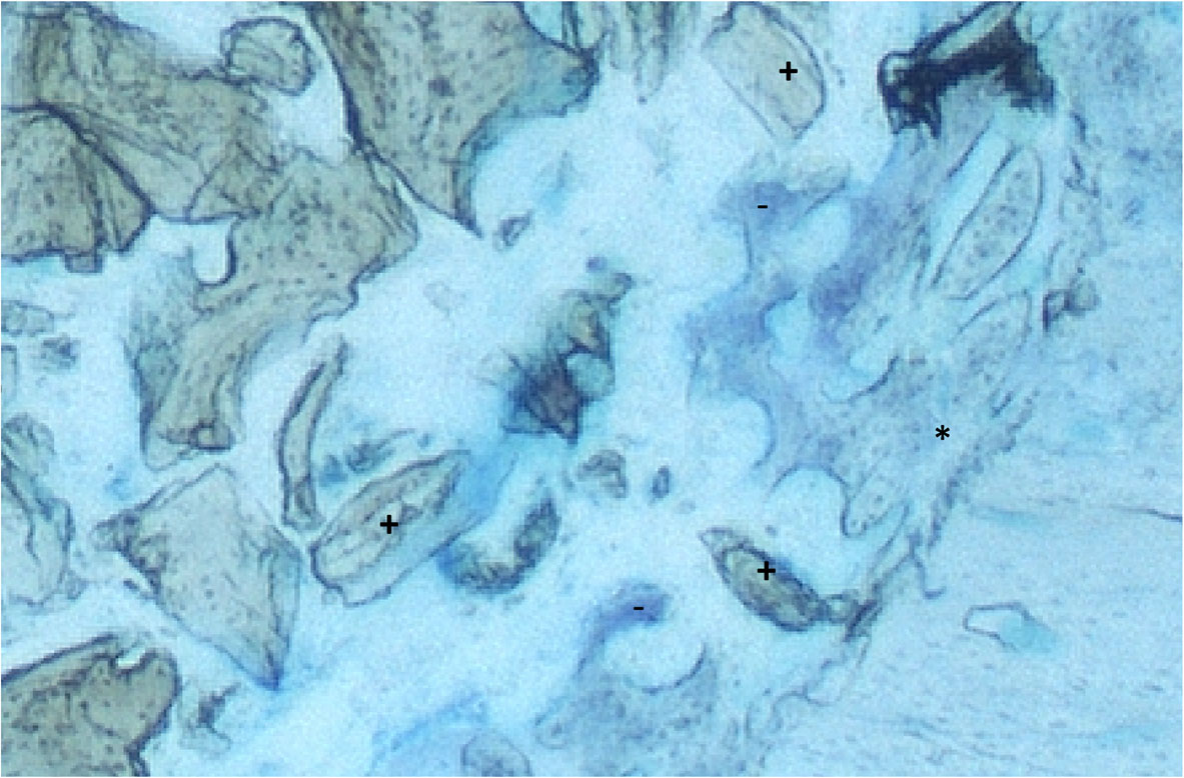
Histology (toluidine blue, original magnification × 20) of a specimen (group with collagen membrane) after 14 days. New osseous growth coming from the residual bone onto the grafted material can be seen. * = residual bone at the edge of the defect; + = bovine bone substitute particles; − = new-formed bone growing from the residual bone

At 24 h, 72 h and 7 days (Fig. 5) after surgery, no new bone formation was seen in both groups. After 14 days, a decent osseous growth coming from the residual bone could be detected, that did not show any significant difference between groups (group “+”: 5.5% (standard deviation: 4.2%), group “-”: 3.3% (standard deviation: 3.1%); *p* = 0.12; Fig. 6). After 3 weeks, there was no significant difference between groups as well (group “+”: 54.4% (standard deviation: 7.7%), group “-”: 49.3% (standard deviation: 5.9%); *p* = 0.25).

## Discussion

To the best of our knowledge, this is the first study that investigated the enzymatic activity in blood and bone after augmentation of bone defects with a bovine bone substitute material, covered or not covered with a collagen membrane. In the early healing phase 24 h after surgery, bone alkaline phosphatase (BAlPh) was significantly elevated in the group that received a collagen membrane. After 72 h, the investigated enzymes bone acid phosphatase (BAcPH) as well as total alkaline phosphatase (TAlPh) and BAlPh were significantly higher in the collagen group. In contrast, in the later healing phases - after two and 3 weeks - all enzymes in the group without a membrane showed higher activity. The porcine collagen membrane consists of a thick bilayer structure resulting in a considerable amount of type I and III collagen that might influence early healing activities. In accordance, the modification of the same xenogenic bone substitute with collagen has already shown to increase initial platelet consumption together with a higher release of VEGF, PDGF and TGF-beta when compared to the bone substitute without collagen adjunct [29]. Also, the superiority of allogenic bone over xenogenic bone substitutes both in pre- as well as clinical studies has been attributed to its incorporated growth factors and collagen [32, 38, 39].

TAcPh and BAcPH are seen as bone resorption marker whereas both TAlPh and BAlPh are assumed to play a role in osteoid formation and bone mineralization [40]. Therefore, the use of a membrane in osseous regeneration procedures of the jaws significantly enhances bone remodeling activity in the early healing phase and accelerates bone regeneration. These results are in accordance to the literature. For example, it could be shown that guided bone regeneration (GBR) procedures result in an increased peri-implant bone growth even if a group without membrane was missing in this study [15]. Turri et al. examined the molecular and structural pattern of bone healing in rat femurs bone defects with and without naturally derived resorbable membranes. In this study, in contrast to our study, histomorphometry showed that the presence of the membrane promoted bone formation in early and late periods. In concordance, upregulation of cell recruitment and coupled bone remodeling genes in the defect were seen. Cells recruited into the membrane expressed signals for bone regeneration like BMP-2, FGF-2, TGF-b1 and VEGF. Western blot and immunohistochemistry analysis demonstrated that the single native membrane contained FGF-2 but not BMP-2. However, an accumulation of FGF-2 and BMP-2 proteins and immunoreactive cells were demonstrated in the implanted membrane in vivo. Though, the authors used a rat femur model without bone substitute materials. In the present study, a full thickness critical size, perforating bone defect [34] reconstructed with a xenogenic bone substitute material was assessed; therefore, the results of Turri et al. cannot be extrapolated to the present study [2]. Several studies have shown that serum and urinary bone turnover markers are able to reflect the healing process depending on the location, type and size of the defect [41]. In an animal study by Komnenou et al., as well as in a study by Singh Ajai et al. in human patients, alkaline phosphatase (AlPh) activity was determined throughout the healing process of fractures. In the group of patients who had a normal bone healing compared to the delayed healing group, significantly higher serum AlPh activity levels were found. The serum levels of AlPh are the sum of the iso-enzymes from the intestine, placenta, liver and bone. The bone AlPh and liver isoforms represent the most relevant fraction of total AlPh activity, with an almost equal contribution to about 95% of this enzyme. In the absence of pregnancy and liver or intestinal disorders, AlPh activity could be a low-cost marker for monitoring the bone fracture healing process [41–43]. Plagnat et al. even suggested that longitudinal monitoring of AlPh in peri-implant crevicular fluid has a potential to be a marker for dental implant failure [44]. In consideration of the change over time of the mean values of the AlPh-level, the AlPh-level decreased in the test group after 1–4 weeks and then increased after 6, 8, 10 and 12 weeks. These results are similar to those of an animal study on gene expression of AlPh during the osseointegration period [45]. However, Tirachaimongkol and colleagues noted no significant differences in AlPh levels over time [46]. Piattelli and colleagues analyzed the histochemical characterization of AlPh and acid phosphatase (AcPh) at the bone-implant interface after the insertion of smooth screw-shaped threaded titanium implants in rabbit tibia [47]. It was found that there is a strong decrease in AlPh activity from the third week. After 2 months it could be noticed that the AlPh and AcPh activities were similar, possibly in terms of bone remodeling.

In the present study, both BAlPh and TAlPh reached their peak after 72 h and rose again between the first and second postoperative week, both in the membrane group and in the non-membrane group. This has been demonstrated in earlier studies as well [23, 48–51]. As for the bone specific isoform BAlPh, Emami et al. demonstrated lower values in patients with delayed healing earlier in the fracture healing process than patients with normal bone union [48].

Though, even if significant differences in bone turnover marker between the groups with and without collagen membrane were seen in our study, this did not seem to influence the formation of new bone in toluidine blue stained histological sections. Lateral wall defects with three to four intact bone walls have a comparable high biological capacity for regeneration [52]. For those defects in the jaws - if the bone substitute material can be stabilized properly - the clinical advantageous effect of barrier membranes could not be proven yet [17, 53]. On contrary, more demanding defects have shown to benefit from GBR-techniques [54], maybe also because of the early increase platelet-derived growth factors [29] in combination with the (later) increase of bone turnover markers as found in the present study. Therefore, a critical size, perforating full thickness defect with limited healing properties [34] was chosen. Even so, for example, at the lingual cortical bone plate, no collagen membrane was applied and therefore no effect can be assumed at this site. In addition, a more elaborated histological analysis including more parameter might be needed that should be focused at in future studies. Also, as there in an increased demand of alloplastic as well as allogeneic materials, these bone substitutes in combination with collagen should be addressed as well.

## Conclusion

Higher levels of enzyme activity indicate a more intense bone remodeling. The results of this study gives hints that GBR with bone substitute particles and collagen membrane show a desirable, significantly earlier bone remodeling activity when compared regeneration procedures with bone substitute particles only. Therefore, the membrane during GBR potentially acts like a bioactive compartment rather than just a passive barrier. Even so, these enzymatic results could not be verified in terms of new bone formation.

## Data Availability

The dataset used and/or analyzed during the current study are available from the corresponding author on reasonable request.

## Abbreviations

GBR: GUIDED bone regeneration
BBSM: Bovine bone substitute material
TAcPh: Total acid phosphatase
BAcPh: Bone acid phosphatase
TAlPh: Total alkaline phosphatase
BAlPh: Bone alkaline phosphatase
AlPh: Alkaline phosphatase
